# Co-Administration of Vitamin E and Testosterone Attenuates The Atrazine-Induced Toxic Effects on Sperm Quality and Testes in Rats

**DOI:** 10.22074/cellj.2016.490

**Published:** 2017-02-22

**Authors:** Hamed Rezaie Agdam, Mazdak Razi, Amir Amniattalab, Hassan Malekinejad, Morteza Molavi

**Affiliations:** 1 Private Veterinary Practitioner, Urmia, Iran; 2 Department of Comparative Histology, Faculty of Veterinary Medicine, Urmia, Iran; 3 Department of Pathology, Faculty of Veterinary Medicine, Islamic Azad University, Urmia Branch, Urmia, Iran; 4 Department of Pharmacology and Toxicology, Faculty of Veterinary Medicine, Urmia, Iran

**Keywords:** Oxidative Stress, Testosterone, Atrazine, Vitamin E, Testis

## Abstract

**Objective:**

Atrazine (ATZ) as a widely used herbicide is considered as a potent endocrine disrupter which adversely affects reproductive systems in both genders. This study aimed to assess the effects of testosterone (T)- and vitamin E (VitE)- alone and their coadministration on testicular function and sperm parameters after exposure to ATZ in rats.

**Materials and Methods:**

In this experimental study, the rats (n=30) are assigned into the following 5 groups: control-sham group (n=6) receiving corn oil, ATZ group (n=6) receiving 200 mg/kg ATZ alone, ATZ+VitE group (n=6) receiving 150 mg/kg ATZ+VitE, ATZ+T group (n=6) receiving 400 µg/kg ATZ+T, and ATZ+VitE+T group (n=6) receiving ATZ+VitE+T for 48 consecutive days. Total antioxidant capacity (TAC), total thiol molecules (TTM), and malondialdehyde (MDA) were analyzed. Serum levels of T, luteinizing hormone (LH), and inhibin-B (IN-B) were also determined. Histological examination and sperm analysis were performed. The data were analyzed using Graph-Pad Prism software version 2.01.

**Results:**

Co-administration of VitE and T significantly (P<0.05) increased ATZ-decreased TAC and TTM levels and reduced ATZ-increased MDA content. T and VitE significantly (P<0.05) increased serum levels of ATZ-reduced T (1.94 ± 0.96), IN-B (122.10 ± 24.33) and LH (0.40 ± 0.10). The T+VitE animals showed a reduction in apoptotic cells and an increase in Leydig cells steroidogenesis. Co-administration of T and VitE significantly (P<0.05) reduced the ATZ-induced DNA disintegrity and chromatin de-condensation. VitE and T protected germinal cells RNA and protein contents against ATZ-induced damages.

**Conclusion:**

T and VitE in simultaneous form of administration were able to normalize the ATZ-induced derangements through promoting antioxidant capacity and endocrine function.

## Introduction

Previous reports have suggested that Atrazine (ATZ, 2-chloro-4-ethylamino-6-isopropyl aminos- triazine), a selective herbicide for inhibiting photosynthesis in broad leaf, grassy weeds, corn, sorghum, sugarcane and cotton has been considered as endocrine disrupter and may have adverse effects on fertilizing potential in both genders of mammalians (1-3). Various findings have indicated that chronic exposure to ATZ potentially resulted in increased sperm abnormality (4-6), delayed gonad maturation (1), arrested spermatogenesis (3, 7) and severe reduction in seminal vesicle and prostate weights (7).

Moreover, chronic exposure to ATZ has been reported to reduce the levels of luteinizing hormone (LH) and testosterone (T) (1, 3, 7). It has been also shown that the ATZ down-regulates the genes participating in steroidogenic activities such as cytochrome P450 aromatase (CYP19A1) (8). Similarly, in a study by Friedmann (9), they have showed that chronic administration of 50 mg/kg of the ATZ for 22 and 48 days inhibited the T production. Previous reports have revealed that the suppression of pituitary hormone after chronic administration of ATZ affected the central nervous system. Accordingly, gonadotropins-releasing hormone (GnRH) secretion was suppressed following oral administration of 100 and 200 mg/kg of ATZ, whereas the single dose administration of GnRH enhanced the LH surge from pituitary in rats (10). In turn, LH stimulates Leydig cells to control Sertoli cells physiologic function by surging T (11).

A number of studies on ATZ-exposed animals have shown the enhancement of germinal cells degeneration in seminiferous tubules increased morphologically abnormal testicular spermatozoa content and polymorphonuclear leukocytes infiltration in testicular tissue (2, 12). Generation of reactive oxygen species (ROS) has been suggested as a mechanism for these changes in testis. In this regard, the oral administration of ATZ at dose levels of 100 and 200 mg/kg resulted in severe oxidative stress in testicular tissue of mature rats. Additionally, chronic exposure to ATZ resulted in a significant increase in percentages of sperms with abnormal chromatin condensation and sperm DNA disintegrity after 48 days (13).

Therefore, ATZ exerts its impact by totally two different mechanisms, meaning a reduction in testicular endocrine and antioxidant status.

Vitamin E (VitE) is a lipid soluble antioxidant which inhibits free radical generation as well as lipid peroxidation in biological systems such as testicular tissue. Indeed, VitE protects spermatogenesis against oxidative stress and enhances male fertilization potential (14). Similarly, Momeni et al. (15) have showed that VitE significantly improved sperm quality and ameliorated the testicular morphologic parameters in sodium arsenite-treated rats. Moreover, it has been reported that VitE improved plasma level of gonadal hormones and enhanced fertilizing capacity in noise-stressed rats (16). We have already shown that co-administrating VitE with T up-regulated the testicular endocrine and antioxidant status (17).

On the other hand, T is required for spermatogenesis progress beyond meiosis and also needed for mature spermatids release during stage VIII in rats. Indeed, the T withdrawal results in a significant derangement in integrity of the blood-testis-barrier (18), conversion of round spermatids to elongated spermatids (19), as well as release of spermatids from Sertoli cells (20).

Therefore, we intended to assess the protective effects of T and VitE-alone and their simultaneous administration on testicular function and sperm parameters after exposure to ATZ in rats.

## Materials and Methods

### Animals

In this experimental study, thirty mature male Wistar rats, 8 weeks old, weighting 200 ± 5 g were used. The rats were obtained from the Animal Resources Center of Islamic Azad University, Urmia Branch, Iran, and were acclimatized in an environmentally controlled room with temperature of 20-23˚C and a 12 hour light/12 hour dark cycle. Food and water were given ad libitum. In this study all experiments conducted on animals were in accordance with the guidance of Ethical Committee for Research on Laboratory Animals of Islamic Azad University, Urmia Branch.

### Experimental design

After one week of acclimation, the animals (n=30) were divided (n=6) into five groups. The animals in control-sham group (n=6) received the same body weight-based volume of the vehicle (corn oil 0.2 ml/day, orally), which was also given to the 4 test groups. ATZ was solved in corn oil and given at the volume of 0.2 ml. The animals in test group were then subdivided into 4 groups that were specifically named based on what they received for 48 days as follows: ATZ, ATZ+VitE, ATZ+T and ATZ+VitE+T. Based on previous study on appropriate doses of VitE and testosterone, all animals received a vehicle including ATZ (daily), VitE (every 48 hours) by gastric gavage and T by intraperitoneal (IP) injection (daily). ATZ, VitE and T were administered at doses of 200 mg/kg (13), 150 mg/kg (21) and 400 μg/kg per body weight (22), respectively.

### Hormone analysis

After 48 days, the animals were anesthetized with ketamine (5%, 40 mg/kg, IP) and xylazine (2%, 5 mg/kg, IP). Blood samples were prepared directly from the heart by letting the blood samples clot at room temperature for 15 minutes before being centrifuged at 3000 g for 10 minutes to obtain the serum. The serum samples were stored at -70˚C for subsequent assays. Serum level of T was analyzed by competitive chemiluminescent immunoassay kit (DRG, Germany and Pishtaz Teb, Iran). The serum concentration of LH was determined by RIA kits using a Biotrak assay system (Amersham Life Science Inc., USA). The serum level of inhibin-B (IN-B) was evaluated by enzyme immunometric assay technique (23).

### Autopsy and organ weight

At the end of experiment, the rats were weighed. The left side of testicular tissues were excised, dissected free from surrounding tissues under the high magnification (×400) provided by a stereo zoom microscope (Olympus, Japan) and the weight was determined. Finally, the testicular to total body weight gains were evaluated in different groups.

### Assessment of total antioxidant capacity

To determine the effect of ATZ on antioxidant status, total antioxidant capacity (TAC) of the testicular tissue was measured. The assessment was carried out based on ferric reduction antioxidant power (FRAP) assay as previously reported (24). Briefly, at low pH, provided by acetate buffer (300 mM, pH=3.6), reduction of ferric tripyridyltriazine (Fe^III^-TPTZ) complex to the ferrous form produces an intensive blue color that is measurable at 593 nm. The intensity of the complex following addition of the appropriate volume of the homogenized tissue to reducible solution of Fe^III^-TPTZ is related to total reducing power of the electron donating antioxidant. For blank and standard solution, the aqueous solution of Fe (FeSO_4_.7H_2_O) and appropriate concentration of freshly prepared ascorbic acid were used, respectively.

### Measurement of serum total thiol molecules

In order to estimate the tissue total thiol molecules (TTM) level, the total sulfhydryl level analyzed in testicular tissue was measured (25). In short, 0.3-0.4 g of the testis samples were homogenized in ice-cold KCl (150 mM), and the mixture was then centrifuged at 3000 g for 10 minutes. About 0.5 ml of the supernatant was added to 0.6 ml Tris-EDTA buffer (Tris base 0.25 M, EDTA 20 mM, pH=8.2) followed by the addition of 40 μl 5,5′-Dithiobis (2-nitrobenzoic acid) (DTNB, 10 mM in pure methanol) in a 10 ml glass test tube. The final volume of the mentioned mixture was made up to 4.0 ml by extra addition of methanol. After incubation for 15 minutes at room temperature, the samples were centrifuged at 3000 g for 10 minutes, and the absorbance of the supernatant was assessed at 412 nm.

### Determination of malondialdehyde

In order to evaluate the lipid peroxidation ratio, the malondialdehyde (MDA) content of the testicular tissues were measured using the thiobarbituric acid (TBA) reaction as described previously (26). About 0.3-0.4 g of the testicular samples were homogenized in ice-cold KCL (150 mM), the mixture was centrifuged at 3000 g for 10 minutes, 0.5 ml of the supernatant was vortexed with 3 ml phosphoric acid (1% v/v), and finally 2 ml of 6.7 g L-1 TBA was added to the samples. The samples were heated at 100˚C for 45 minutes and then cooled on ice. Finally, 3 ml N-butanol was added and the samples were further centrifuged at 3000 g for 10 minutes. The absorbance of supernatant was analyzed using spectrophotometer at 532 nm. The MDA concentration was calculated according to prepared calibration curves using MDA standards. The results for MDA were expressed as nmol per mg protein of the samples. The protein content of the samples was measured according to the Lowry method (27).

### Evaluation of epididymal sperm characteristics

The epididymis was dissected off of the testicles under a stereo zoom microscope (magnification: ×20, Olympus, Japan). The epididymis was divided into 3 segments as follows: caput, corpus and cauda. The caudal epididymal tissue was trimmed and minced in 5 mL Hams F10 medium. After 20 minutes, the released spermatozoa from the epididymal tissue were transferred into a new tube. The sperm count was performed according to the standard hemocytometric lam method (28). Eosin-nigrosin staining was performed to analyze the sperm viability. The sperms with stained head piece were considered as dead sperms. For this purpose, 20 smeared slides were prepared from each sperm sample of animals from different groups. The percentage of dead sperms (sperms with stained cytoplasm) was compared among groups.

### Assessment of sperms chromatin condensation

The aniline-blue staining was performed to evaluate sperm chromatin condensation (29). Briefly, after sperm preparation, 5 μl of the prepared spermatozoa were spread onto glass slides and allowed to dry. The smears were fixed in 3% buffered glutaraldehyde in 0.2 M phosphate buffer saline (PBS, pH=7.2) for 30 minutes. Slides were then stained with 5% aqueous Aniline Blue and mixed with 4% acetic acid (pH=3.5) for 5 minutes. About 100 sperm cells per slide were analyzed and the percentage of unstained sperm heads was calculated.

### Acridine orange staining for sperm DNA strands

Acridine orange (AO) staining was performed to estimate the sperm DNA fragmentation (30). In brief, air dried slides were stained for 10 minutes with freshly prepared AO (0.19 mg/ml) and washed in distilled water, while the coverslip was applied on the slides. The slides were evaluated on the same day using an epi-fluorescent microscope (Nikon, Japan). In all preparations, at least 100 spermatozoa were evaluated at ×400 magnification. Spermatozoa with green fluorescence were marked to have native double strand DNA (DS-DNA), the yellowish fluorescence stained spermatozoa were considered as having partly denatured single-stranded-DNA (PSS-DNA), and red fluorescence stained were considered as completely denatured single-stranded-DNA (SS-DNA). Percentage of green, yellow and red spermatozoa were evaluated and compared among groups.

### Histological analysis

Dissected tissue samples were washed with distilled water and fixed in Bouin’s fixative before histological analysis. The testicular samples were paraffin embedded and cut (5-6 μm) by a rotary microtome (Microm Labogeräte GMBH, Germany). The sections (5-6 μm) were stained with Iron-Weigert (Pajohesh Asia, Iran) for detection of nuclei of germinal epithelium in the testis. The prepared slides were analyzed under a light microscope by multiple magnifications (×400 and ×1000). The numbers of Leydig cells per one mm^2^ of interstitial connective tissue and Sertoli cells per one seminiferous tubule were numerated. The percentage of seminiferous tubules with more than 3-4 germinal layers and percentage of tubules with normal spermiogenesis were considered as positive tubular differentiation index (TDI) and positive spermiogenesis index (SPI), respectively. The percentage of tubules with positive repopulation index (RI), as the ratio of active spermatogonia (spermatogonia type B with light nucleus using Iron-Weigert staining technique) to inactive spermatogonia (spermatogonia type A with dark nucleus using Iron-Weigert staining), was calculated. In order to evaluate the Sertoli cells distribution in one seminiferous tubule, the periodic acid-Schiff (PAS) staining was performed. Two hundred tubules from 20 sections were analyzed for each sample and the results were reported as the number of Sertoli cells per one tubule to compare among groups.

### Histochemical analysis for Leydig cells steroidogenic activity

In order to count the Leydig cells, the Sudan Black B staining was performed using a commercial kit (Science, Iran). In order to support the histochemical analysis, the fluorescent staining for steroidogenic compounds was performed using a special kit (FLP, IUO100, Pajohesh Asia, Iran). In brief, the frozen sections were hydrated and treated with iron-free aluminum sulfate (Alum) solution for 10 minutes. The slides were then washed with distilled water that was followed by being stained with a special fluorescent dye (FITC-conjugated1-anilinonaphthalene-8-sulphonate) for steroidogenic foci for 5 minutes. After being rinsed with deionized water, the slides were blotted with a fluorescent mountant. In order to reduce the bias problems, 20 sections for each sample were investigated, and the Leydig cells distribution per one mm^2^ of the interstitial connective tissue was reported.

### Histological analysis of apoptosis using TUNEL assay

Terminal deoxynucleotidyl transferase (dUTP) nick-end-labeling (TUNEL) technique was performed to detect the in situ DNA fragmentation (31). In brief, the deparaffinized tissues were predigested with 20 ml proteinase K for 20 minutes and incubated in PBS solution containing 3% H_2_O_2_ for 10 minutes to block the endogenous peroxidase activity. In order to detect in situ cell death, the sections were incubated with the TUNEL reaction mixture (Roche, Germany) for 6 minutes at 37˚C. The slides were then rinsed three times with PBS and incubated with secondary anti-fluorescein-POD-conjugate (Sigma-Aldrich, Germany) for 30 minutes. After washing three times with PBS, diaminobenzidine-H_2_O_2_ (DAB) substrate chromogenic solution (Roche, Germany) was added on sections. For negative control, TUNEL reaction mixture was replaced with nucleotide mixture in reaction buffer. The total and apoptotic Leydig cells distribution per one mm2 of the interstitial tissue and total normal as well as apoptotic Sertoli cells number per 20 seminiferous tubules in 5 sections of one specimen evaluated and reported. The numbers of normal and apoptotic spermatogonia cells per one seminiferous tubule were found by evaluation of five sections of twenty tubules for each sample.

### Fluorescent analysis of RNA damage

The RNA damage was assessed based on Darzynkiewicz method (32). In brief, the testes were washed with ether alcohol and cut by cryostat (8 μm). The prepared sections were fixed by different degrees of ethanol for 15 minutes, briefly rinsed in 1% aqueous acetic acid and finally washed in distilled water. The specimens were stained with AO (Sigma-Aldrich, Germany) for 3 minutes, distained in PBS, and then analyzed for fluorescent colors differentiation in calcium chloride. The degenerated germinal cells were characterized by loss of RNA and/or with faint red stained RNA. The normal cells were marked with bright red RNA at the apex of the nucleolus. Bias problems for staining density were reduced by analyzing 20 sections for each sample.

### Statistical analysis

All data were presented as mean ± SD. Results were analyzed using Graph-Pad Prism software version 2.01 (Graph Pad software Inc., USA). The comparisons between groups were made using one way analysis of variance (ANOVA) and Bonferroni post-hoc test. A P<0.05 was considered significant.

## Results

### General observations

**Fig.1:**
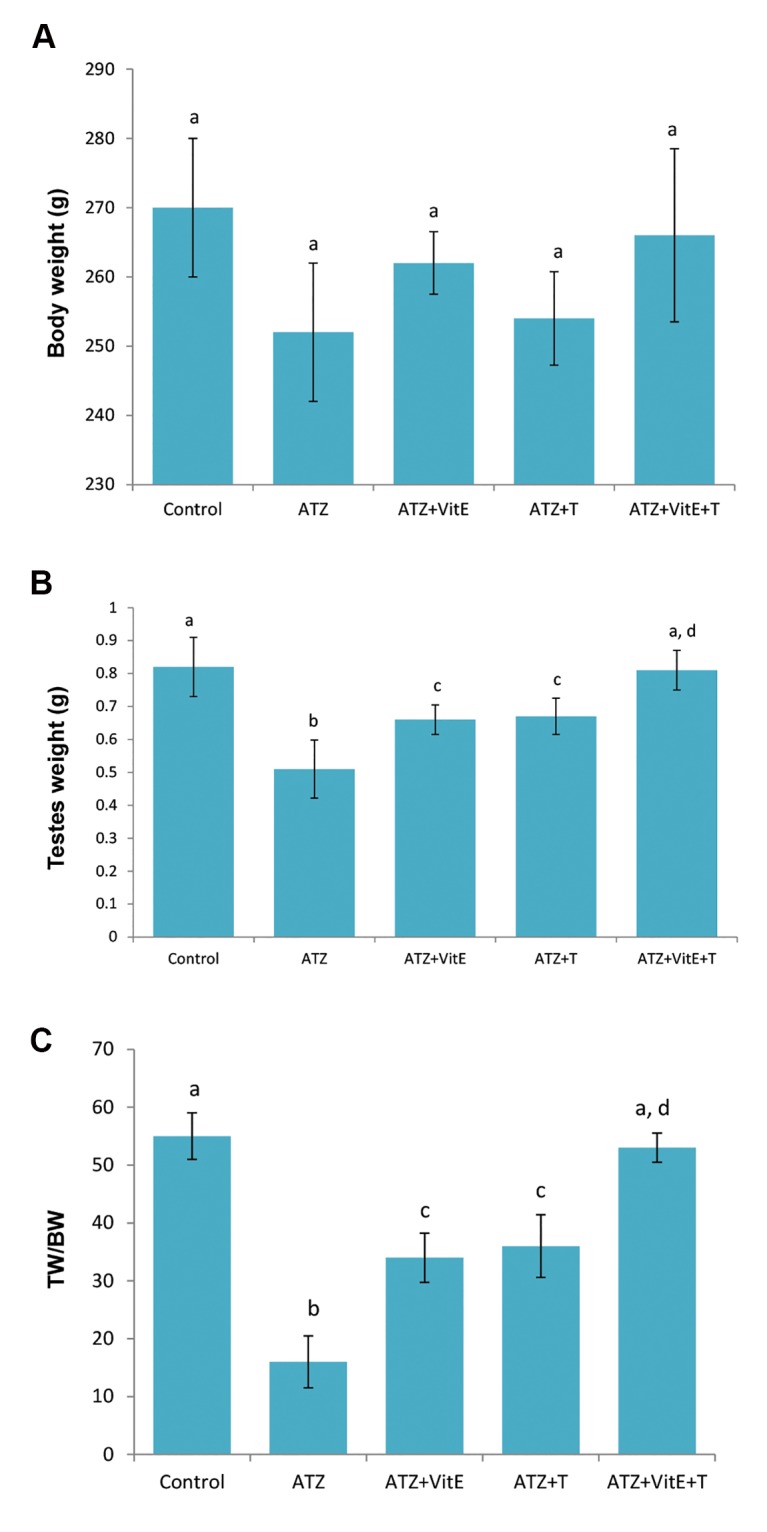
General alterations of total body weight, testicular weight and testicular weight relative to total body weight, A. Effects of VitE (150 mg/kg) and T (400 μg/kg) alone and their simultaneous administration on ATZ-induced (200 mg/kg) changes on total body weight, B. Testicular weight, and C. Testicular weight to body weight ratio. All data are presented in mean ± SD. a vs. b; Significant at P<0.05, c and d vs. b; Significant at P<0.05, ATZ; Atrazine, VitE; Vitamin E, T; Testosterone, TW; Testicular weight, and BW; Body weight.

After 48 days, body weight of the ATZ-exposed animals decreased compared to the control-sham group, but it was not statistically significant. Accordingly, animals in the VitE+T group revealed the highest body weight gain. The testicular to body weight ratio remarkably declined in the ATZ-exposed animals as compared to the VitE+T and control-sham groups (Fig .1A-C).

### Vitamin E and testosterone enhanced the Atrazine-declined serum levels of Luteinizing hormone, testosterone and Inhibin-B levels

The animals in ATZ-exposed group exhibited a significant (P<0.05) decrease in serum levels of LH, T and IN-B as compared to the VitE+T group. The animals in the VitE+T group showed the highest concentration of T and IN-B in comparison to those treated with T VitE alone. Co-treatment of T with ATZ did not change the serum level of LH, while LH concentration elevated after treatment with VitE+T and VitE alone. The data for hormonal changes are presented in Table 1.

### Vitamin E and testosterone improved the Atrazine-induced antioxidant status

The results of biochemical analysis demonstrated that both TAC and TTM levels significantly (P<0.05) decreased after exposure to ATZ. In contrast, administration of VitE and T remarkably (P<0.05) increased the TAC and TTM levels in comparison to ATZ-exposed animals. Surprisingly, the TAC elevation in ATZ+VitE+T group (0.62 ± 0.02 μmol/ml) was higher than that of the ATZ+VitE (0.54 ± 0.04 μmol/ml) and ATZ+T (0.44 ± 0.03 μmol/ml) groups. The ATZ-exposed group showed significant increase in testicular tissue MDA level as compared to the control-sham animals. Meanwhile, T and VitE co-administration remarkably (P<0.05) decreased tissue MDA level. Accordingly, the ATZ+VitE+T- group evinced the lowest MDA level in comparison to ATZ+T and ATZ+VitE groups (Table 1).

### Vitamin E and testosterone enhanced sperm quality

**Fig.2:**
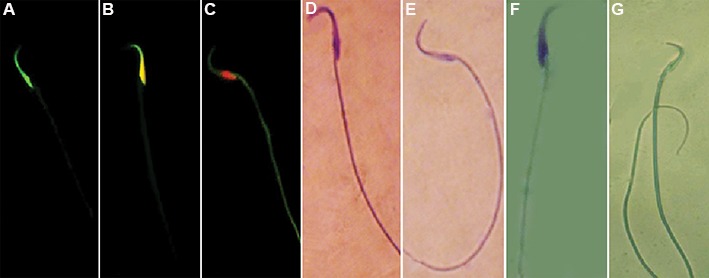
Photomicrograph of sperms, A. Sperm with normal DS-DNA, B. Sperm with PSS-DNA, C. Sperm with SS-DNA, D. Dead sperm with stained cytoplasm, E. Live sperms with colorless cytoplasm, F. Sperm with immature ChC, and G. Sperm with ChC. A, B, C. AO staining, D, E. Eosin-nigrosin staining, F and G. Aniline blue staining. AO; Acridine orange, DS-DNA; Double strand DNA, PSS-DNA; Partly single stranded DNA, SS-DNA; Single strand DNA, and ChC; Chromatin condensation.

The animals in ATZ-exposed group showed a significant (P<0.05) reduction in sperm count and viability compared to the control-sham group. Moreover, the percentage of sperms with condensed chromatin and DS-DNA significantly (P<0.05) reduced in the ATZ-exposed animals. Our findings showed that both VitE and T at the studied doses prevented significantly (P<0.05) the ATZ-induced chromatin decondensation and DNA damage. The animals in ATZ+VitE+T group showed an increase in sperm count, viability, chromatin condensation and DNA integrity in comparison to the ATZ+T and ATZ+VitE groups (Fig .2A-G, Table 2).

### Vitamin E and testosterone inhibited the Atrazine impact on germinal cells

Histological observations demonstrated that the animals in the ATZ-exposed group showed highly degenerated testes with remarkable seminiferous tubules atrophy accomplished by severe edema in interstitial tissue. Meanwhile, the seminiferous tubules atrophy and edema remarkably reduced in the ATZ+VitE+T group. The animals in the ATZ+VitE+T group revealed an improvement in histomorphometric features such as higher thickness of germinal epithelium and higher percentage of tubules with positive TDI, RI and SPI. Light microscopic analysis indicated that the ATZ+T+VitE group showed the highest number of Sertoli cells per one tubule as compared to the ATZ-exposed group (Fig . 3A1-Fig E1, F). The results from histomorphometric analysis are presented in Table 3. TUNEL assay was performed to evaluate the number of apoptotic Sertoli, Leydig and spermatogonia cells. Observations revealed that the simultaneous administration of VitE and T significantly (P<0.05) reduced the ATZ-induced apoptotic cells (Fig . 3A2-Fig E2). The data for cellular apoptosis are presented in Table 4.

### Vitamin E and testosterone improved Leydig cells steroidogenic activity

Hypertrophied Leydig cells with granulated cytoplasm were revealed in the ATZ-exposed animals. The ATZ-exposed rats showed a significant (P<0.01) increase in the percentage of Leydig cells with faint lipophilic cytoplasm as compared to the control-sham group. Moreover, the number of Leydig cells per one mm^2^ of the connective tissue significantly (P<0.05) deceased in the ATZ-exposed rats. By contrast, Leydig cells distribution and intracytoplasmic steroid accumulation increased, whereas number of hypertrophied Leydig cells decreased in the ATZ+VitE+T animals. The data for Leydig cells distribution is presented in Table 5.

The fluorescent staining for Leydig cells intracytoplasmic steroidogenic foci supported the histochemical analysis. Remarkably (P<0.05) higher numbers of Leydig cells with steroid synthesis were observed in the ATZ+VitE and ATZ+VitE+T groups (Fig . 4A-E).

### Vitamin E and testosterone reduced the Atrazine- induced RNA damage

Fluorescent analysis for RNA damage in testicular tissue showed significantly (P<0.05) higher percentage of seminiferous tubules with damaged RNA content in germinal cells. By contrast, co-administration of VitE and T significantly (P<0.05) reduced the ATZ-induced RNA damage (Fig . 4A-E). The statistical analysis showed a positive correlation between the percentages of tubules with RNA damage and testicular total protein level (Figes. 5A-C, 6).

**Table 1:** Effects of T and VitE on ATZ-induced changes in serum levels of T, IN-B, LH, TAC, TTM and MDA


Group	T (ng/mL-1)	IN-B (pg/mL-1)	LH (ng/mL-1)	TAC (μmol/mL)	TTM (μm/mL)	MDA (nmol/mL)

Control-sham	5.85 ± 0.63^a^	342.00 ± 10.48^a^	2.56 ± 0.44^a^	0.66 ± 0.025^a^	0.17 ± 0.002^a^	1.81 ± 0.076^a^
ATZ	1.94 ± 0.96^b^	122.10 ± 24.33^b^	0.40 ± 0.10^b^	0.32 ± 0.021^b^	0.07 ± 0.003^b^	4.06 ± 0.055^b^
ATZ+VitE	3.38 ± 0.33^c^	173.00 ± 8.18^c^	1.11 ± 0.19^c^	0.54 ± 0.042^c^	0.14 ± 0.009^c^	1.83 ± 0.271^c^
ATZ+T	4.35 ± 0.22^d^	266.66 ± 25.16^d^	0.45 ± 0.20^b^	0.44 ± 0.038^d^	0.11 ± 0.015^d^	2.10 ± 0.100^c^
ATZ+VitE+T	5.03 ± 0.70^d^	273.33 ± 15.27^e^	1.04 ± 0.21^c^	0.62 ± 0.028^d^	0.16 ± 0.003^e^	1.75 ± 0.217^c^


All data are presented in mean ± SD. ^a^ vs. ^b^ ; Significant at P<0.05, ^c, d^ and ^e^ vs. ^b^; Significant at P<0.05, ATZ; Atrazine, VitE; Vitamin E, T; Testosterone, IN-B; Inhibin-B, LH; Luteinizing hormone, TAC; Total antioxidant capacity, TTM; Total thiol molecules, and MDA; Tissue malondialdehyde.

**Table 2:** Protective effects of VitE and T on ATZ-induced degeneration on sperm parameters


Parameter	Control-sham	ATZ	ATZ+VitE	ATZ+T	ATZ+VitE+T

DS-DNA (%)	93.75 ± 2.69^a^	54.00 ± 4.08^b^	62.12 ± 2.16^c^	68.50 ± 1.73^d^	81.25 ± 4.85^e^
PSS-DNA (%)	1.50 ± 1.29^a^	31.25 ± 2.21^b^	21.00 ± 2.94^c^	19.50 ± 1.28^c^	14.87 ± 1.57^d^
SS-DNA (%)	0.50 ± 0.57^a^	21.00 ± 1.12^b^	17.50 ± 1.29^c^	16.35 ± 2.17^c^	10.00 ± 0.81^d^
ChC (%)	84.50 ± 3.10^a^	52.75 ± 4.42^b^	62.74 ± 2.21^c^	68.75 ± 2.98^d^	76.73 ± 2.50^e^
Sperm count (×10^6^)	62.25 ± 2.63^a^	37.00 ± 2.44^b^	47.50 ± 3.31^c^	51.25 ± 2.21^c^	57.75 ± 1.70^d^
Viability (%)	90.00 ± 2.94^a^	58.01 ± 3.55^b^	68.75 ± 3.77^c^	66.50 ± 3.00^c^	88.00 ± 3.26^d^


All data are presented in mean ± SD and %. ^a^ vs. ^b^; Significant at P<0.05, ^c, d^ and ^e^ vs. ^b^; Significant at P<0.05, ATZ; Atrazine, VitE; Vitamin E, T; Testosterone, DS-DNA; Double strand DNA, PSS-DNA; Partly single stranded DNA, SS-DNA; Single strand DNA, and ChC; Chromatin condensation.

**Fig.3:**
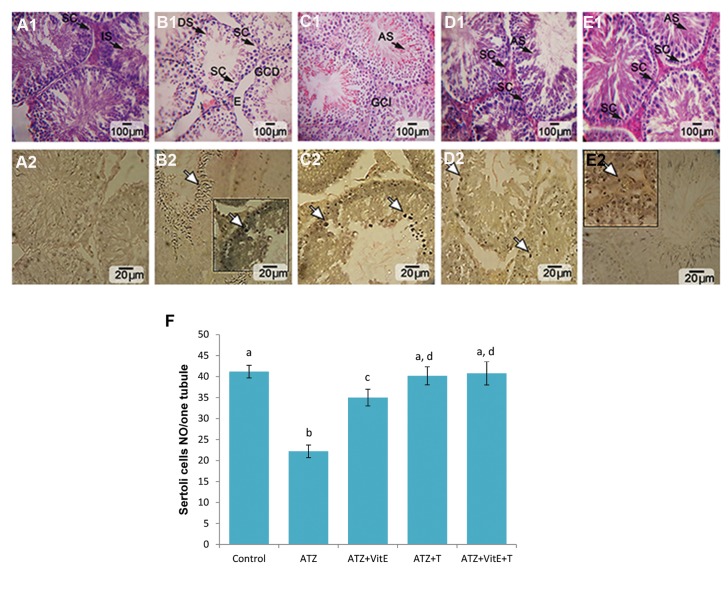
Cross section from seminiferous tubules, A. Control, B. ATZ alone, C. ATZ+VitE, D. ATZ+T, E. ATZ+VitE+T. A1. Representing intact spermatogenesis (IS) in control group with no apoptosis, A2, B1. Note germinal cells dissociation (GCD), edema (E), damaged spermiogenesis (DS) and decreased Sertoli cells (SC) in ATZ-received group, representing intensive apoptosis in B2, C1. See germinal cells integrity (GCI) and ameliorated spermiogenesis in ATZ+VitE-received group with decreased apoptosis at germinal cells level, C2, D1. Note normal Sertoli cells distribution (SC) and ameliorated spermiogenesis (AS) in ATZ+T-received group, representing diminished apoptosis D2, E1. Represents normal Sertoli cells distribution, ameliorated spermatogenesis and spermiogenesis ratio associated with diminished apoptosis E2. H&E and TUNEL Stainings. F. Effects of VitE- and T-alone and their simultaneous administration on ATZ-reduced number of Sertoli cells per one seminiferous tubule. All data are presented in mean ± SD. ^a^ vs. ^b^; Significant at P<0.01, c vs. b; Significant at P<0.01 and d vs. b and c; Significant at P<0.05, ATZ; Atrazine, VitE; Vitamin E, T; Testosterone and TUNEL; Terminal deoxynucleotidyl transferase (dUTP) nick-end-labeling.

**Table 3:** Effects of T and VitE on ATZ-induced negative TDI, SPI, and RI, as well as ATZ-decreased germinal epithelium height


Group	Positive TDI (%)	Positive SPI (%)	Positive RI (%)	GEH (µm)

Control-sham	84.42 ± 6.78^a^	81.32 ± 7.76^a^	76.23 ± 10.12^a^	234.53 ± 23.32^a^
ATZ	45.38 ± 4.64^b^	34.15 ± 10.11^b^	30.42 ± 7.41^b^	112.42 ± 32.41^b^
ATZ+VitE	60.41 ± 7.42^c^	54.11 ± 3.66^c^	56.76 ± 7.45^c^	167.54 ± 12.50^c^
ATZ+T	64.15 ± 4.33^c^	66.30 ± 4.52^d^	64.15 ± 4.33^c^	196.41 ± 18.14^c^
ATZ+VitE+T	78.30 ± 4.53^d^	78.21 ± 5.24^e^	76.41 ± 4.11^d^	200.10 ± 9.72^c^


All data are presented in mean ± SD. ^a^ vs. ^b^; Significant at P<0.05, ^c, d^ and ^e^ vs. ^b^; Significant at P<0.05, ATZ; Atrazine, VitE; Vitamin E, T; Testosterone, TDI; Tubular differentiation index, SPI; Spermiogenesis index, RI; Repopulation index, and GEH; Germinal epithelium height.

**Table 4:** Effects of T and VitE on ATZ-induced cellular apoptosis


	Control-sham	ATZ-induced	ATZ+VitE	ATZ+T	ATZ+VitE+T

Total Sertoli cells	41.33 ± 1.52^a^	22.64 ± 1.22^b^	35.00 ± 2.01^c^	40.37 ± 2.11^a^^,^^d^	40.70 ± 2.56^d^
Apoptotic Sertoli cells	1.38 ± 0.42^a^	17.25 ± 0.95^b^	13.00 ± 2.16^c^	12.75 ± 0.95^c^	7.25 ± 1.70^d^
TSE/TASE (%)	3.39	76.1	34.1	31.58	17.81
Total Leydig cells	35.33 ± 2.08^a^	18.33 ± 1.52^b^	30.64 ± 2.08^c^	29.66 ± 1.52^c^	35.58 ± 1.43^d^
Apoptotic Leydig cells	0.90 ± 0.27^a^	10.75 ± 0.95^b^	8.25 ± 0.50^c^	8.86 ± 0.27^c^	5.03 ± 0.76^d^
TL/TAL (%)	2.5	58.6	26.6	27.81	15.4
Total spermatogonia cells	51.75 ± 4.99^a^	33.75 ± 2.62^b^	40.82 ± 1.96^c^	38.25 ± 1.06^c^	45.50 ± 2.64^d^
Apoptotic spermatogonia cells	2.00 ± 1.41^a^	26.25 ± 1.70^b^	18.75 ± 0.95^c^	21.00 ± 2.00^c^	12.14 ± 1.82^d^
TS/TAS (%)	3.8	77.7	46.01	54.9	26.68


All data are presented in mean ± SD and %. ^a^ vs. ^b^; Significant at P<0.05, ^c, d^ vs. ^b^; Significant at P<0.05, ATZ; Atrazine, VitE; Vitamin E, T; Testosterone, TSE; Total Sertoli cells, TASE; Total apoptotic Sertoli cells, TL; Total Leydig cells, TAL; Total apoptotic Leydig cells, TS; Total spermatogonia cells, and TAS; Total apoptotic spermatogonia cells.

**Table 5:** Effects of T and VitE on ATZ-induced damages on Leydig cells


Group	Leydig cells	H. Leydig cells	H. Leydig cells/Leydig cells (%)

Control-sham	35.33 ± 2.08^a^	-	0^a^
ATZ	18.33 ± 1.52^b^	7.42 ± 1.21	40^b^
ATZ+VitE	30.64 ± 2.08^c^	4.30 ± 1.86	14^c^
ATZ+T	29.66 ± 1.52^c^	-	0^d^
ATZ+VitE+T	35.58 ± 1.43^d^	-	0^d^


All data are presented in mean ± SD and %. ^a^ vs. ^b^; Significant at P<0.05, c, d vs. b; Significant at P<0.05, ATZ; Atrazine, VitE; Vitamin E, T; Testosterone, and H. Leydig cells; Hypertrophied Leydig cells.

**Fig.4:**
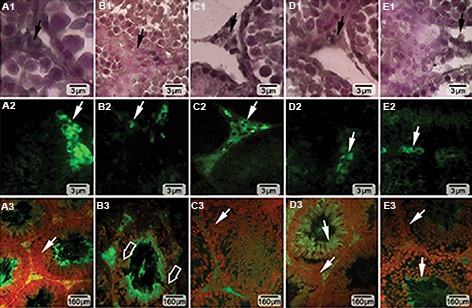
Cross section from seminiferous tubules of A. Control, B. ATZ, C. ATZ+VitE, D. ATZ+T, and E. ATZ+VitE+T groups. First and second rows show intra-cytoplasmic steroid (unsaturated fatty acid) foci in Leydig cells (arrows), which are marked in A1, B1, C1, D1, E1. Black using Sudan Black B, A2, B2, C2, D2, E2. Special flourescent stainig techniques. Alone- and Co-administration of VitE and T enhanced steroidogenesis in Leydig cells, and A3, B3, C3, D3, E3. Represent mRNA damage at germinal cells level. Severe mRNA damage is shown with thick arrows in ATZ-induced group, but it is ameliorated (thin arrows) in ATZ+VitE and ATZ+T groups. ATZ; Atrazine, VitE; Vitamin E, and T; Testosterone.

**Fig.5:**
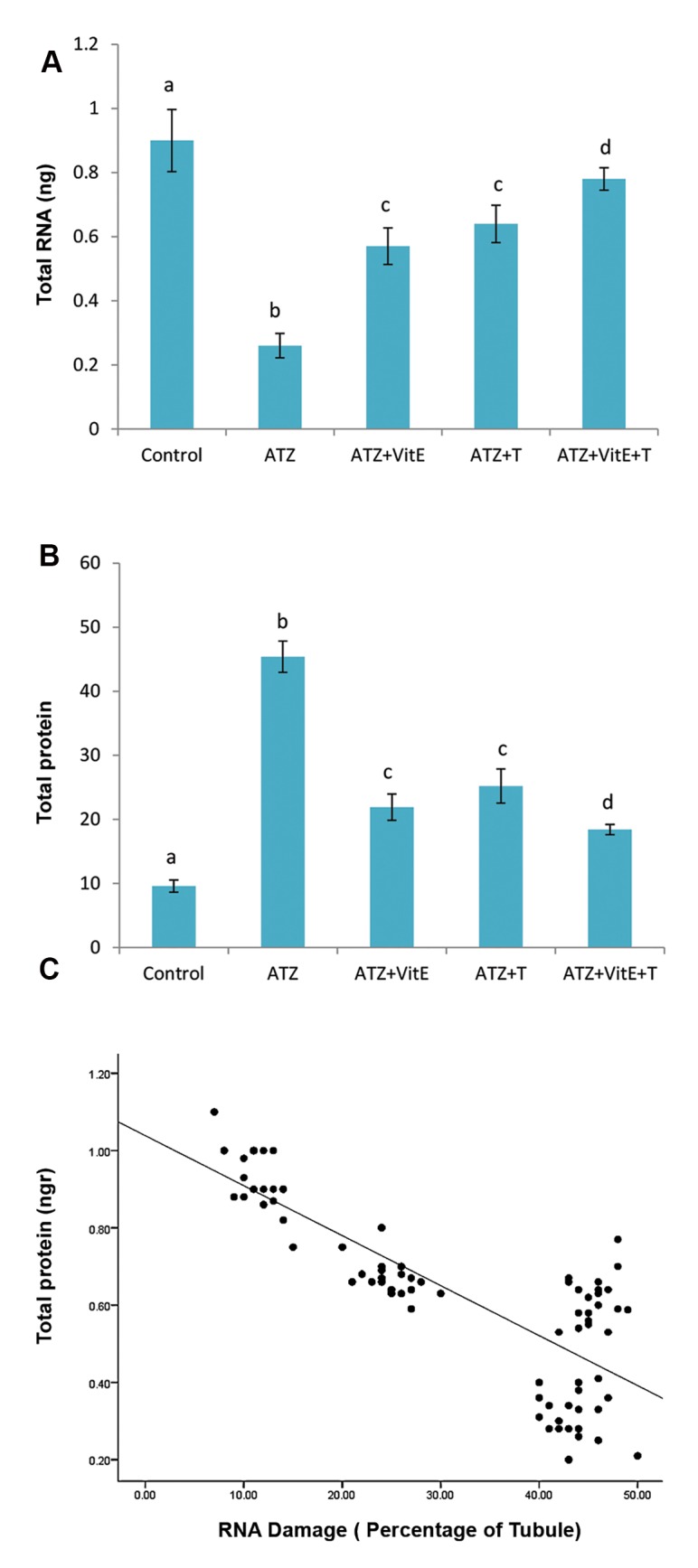
Ameliorative effect of VitE and T in alone and simultaneous forms on mRNA and protein contents, A. Effects of VitE- and T-alone and their simultaneous administration on ATZ-induced RNA damages, B. Decreased total protein content, and C. Positive correlation between percentage of tubules with damaged RNA in germinal cells and testicular total protein content (c), r=0.875, all data are presented in mean ± SD. a vs. b; Significant at P<0.05, c and d vs. b; Significant at P<0.05, ATZ; Atrazine, VitE; Vitamin E, and T, Testosterone.

**Fig.6:**
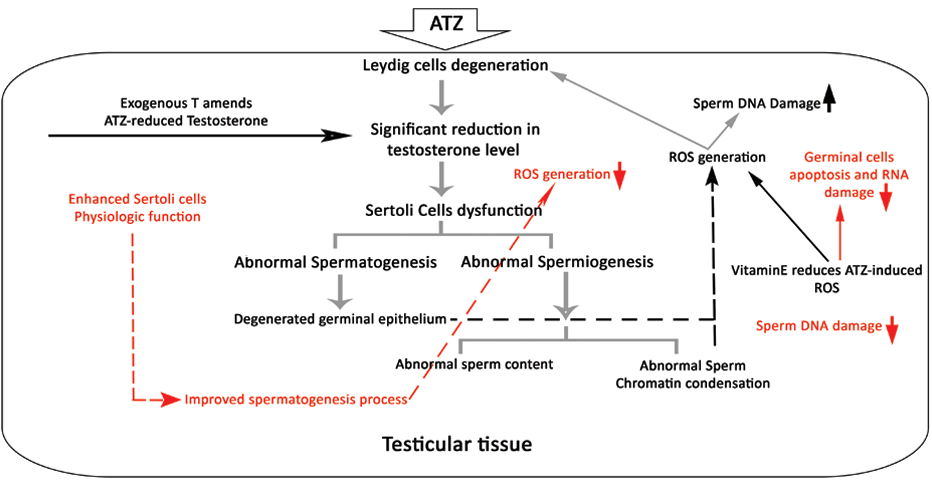
The pathological impact of ATZ on testicular tissue and the protective effects of VitE and T, exogenous T promoted the testicular endocrine system, and VitE reduced the ATZ-induced oxidative stress by up-regulating the antioxidant capacity of the testis. Therefore, with completely different mechanisms, VitE and T reduced ATZ-induced damages. ATZ; Atrazine, VitE; Vitamin E, and T, Testosterone.

## Discussion

Our findings showed that ATZ either by disrupting endocrine which was demonstrated by T and IN-B reduction or by increasing oxidative stress declined spermatogenesis and sperm quality parameters. Additionally, we found that T and VitE-alone and in combination regimens remarkably diminished the ATZ-induced hormonal, biochemical and structural damages. As a primary finding, we showed that administration of ATZ decreased total body and testis weights, but reduction in testis weight was substantial that may attribute to adverse effects of ATZ.

It has been reported that administration of 200 mg/kg of ATZ reduced the T secretion (2, 4). Therefore, in present study, we evaluated the possible restoration of Sertoli cells physiologic activities in the ATZ-exposed rats with exogenous testosterone. The observations demonstrated that the animals in VitE- and T-treated groups showed increased serum levels of T, while the T levels were more distinctive in animals simultaneously receiving VitE and T. This finding suggested that co-administration of VitE and T might have synergistic effects, albeit by different mechanisms. The observed synergistic effects due to coadministration of VitE and T are most likely to be related to the facts that in addition to exogenous T supplementation, VitE both protected Leydig cells from ATZ-induced oxidative stress and supported further Sertoli cells function and other endocrine activities. Microscopes analysis also showed VitE significantly increased intracytoplasmic steroid accumulation in Leydig cells. Considering the importance of intracytoplasmic steroid foci in androgen biosynthesis, increased serum levels of T may attribute to intact Leydig cells function.

The biochemical analysis also showed that the serum levels of LH significantly decreased in the ATZ-exposed animals, but it elevated in ATZ+VitE groups. Surprisingly, it remained unchanged in the ATZ+T rats. One may note that ATZ influenced testicular endocrine activity by reducing the individual Leydig cells ability to produce testosterone, decreasing Leydig cells number and influencing negatively the pituitary-gonadal axis. Therefore, the separate treatment with T did not exert significant changes in LH level. Meanwhile, VitE by ameliorating the LH secretion from pituitary gland enhanced the Leydig cells potential to produce testosterone. On the other hand, VitE protected the Leydig cells against the ATZ-induced derangement via provoking the antioxidant status. Thus, it may be concluded that T and VitE may ameliorate the ATZ-reduced serum level of T with different mechanisms of action.

Indeed, Leydig cells control the Sertoli cells partly by secretion of T (11, 33), and the Sertoli cells play a major role in regulating spermatogenesis (RI and TDI) and spermiogenesis and in controlling the rate of spermatozoa production (34, 35). The Sertoli cells provide structural and nutritional support for germ cells and influence mitotic activities of spermatogonia (36, 37). Our analysis showed that co-treatment with VitE and T significantly improved the ATZ-induced spermatogenesis arrest, abnormal spermiogenesis and defective sperm production. The results indicated that co-administration of these compounds could protect Sertoli cells via ameliorating T level. This conclusion was confirmed by decreased Sertoli cells apoptosis, enhanced serum level of IN-B as well as increased percentage of tubules with positive RI, TDI and SPI in the ATZ+VitE+T- group.

There is a clear correlation between increased oxidative stress with precursor germinal cells apoptosis and sperm DNA damage (38, 39). A number of studied have indicated that after exposure of rats to 100, 200 and 300 mg/kg ATZ, sperm quality was significantly reduced. Sertoli cells also have a major role in sperm morphology and early nuclear maturity such as chromatin packing (3, 39). We found that the co-administration of VitE and T enhanced the sperm parameters by protecting the Sertoli cells against ATZ-induced derangement, albeit by two different mechanisms. Furthermore, VitE enhanced the testicular antioxidant status, which in turn resulted in protecting Sertoli cells against the ATZ-induced oxidative stress. However, administration of exogenous T provoked Sertoli cells physiological activation, meaning that it played the same role as natural T in intact condition. The biochemical findings for testicular TAC, TTM and MDA were in accordance with this hypothesis. Moreover, elevated quality of sperm parameters improved Sertoli cells physiologic function. In correlation with our findings, other studies have showed that VitE at dose level of 100-200 mg/kg significantly reduced the MDA level and promoted the testicular antioxidant capacity (40, 41). It has been also reported that administrating of exogenous T enhanced the testicular endocrine status and provoked the spermatogenesis (42, 43).

Indeed, the haploid germinal cells represent intact mRNA for up to 7 days. The special RNA-binding proteins are involved in maintaining the stability of mRNA through recognition of a specific nucleotide sequences (44). Considering that any damages to cellular RNA and protein contents play essential roles in promoting apoptosis, we may suggest that the VitE by enhancing the antioxidant capacity and T by provoking the endocrine system significantly reduced the ATZ-induced oxidative stress, which in turn enhanced the RNA stability in haploid cells. In this regard, the animals in VitE plus testosterone-administered group showed both higher testicular total protein content and significantly higher percentage of tubules with normal RNA content in different layers of germinal epithelium.

## Conclusion

According to our results, ATZ exerted its pathological effect both by disrupting the endocrine system and by declining the antioxidant power. Also, we found that the co- administration of VitE and T could protect the ATZ-induced derangement by promoting testicular endocrine status and by enhancing the antioxidant capacity, which in turn resulted in protecting germinal cell’s DNA, RNA and protein contents.

## References

[1] Trentacoste SV, Friedmann AS, Youker RT, Breckenridge CB, Zirkin BR (2001). Atrazine effects on testosterone level and androgen-dependent reproductive organs in prepubertal male rats. J Androl.

[2] Victor-Costa AB, Bandeira SM, Oliveira AG, Mahecha GA, Oliveira CA (2010). Changes in testicular morphology and stroidogenesis in adult rats exposed to atrazine. Reprod Toxicol.

[3] Feyzi-Dehkhargani S, Shahrooz R, Malekinejad H, Sadrkhanloo RA (2012). Atrazine in sub-acute exposure results in sperm DNA disintegrity and nuclear immaturity in rats. Vet Res Forum.

[4] Abarikwu SO, Adesiyan AC, Oyeloja TO, Oyeyemi MO, Farombi EO (2010). Changes in sperm characteristics and induction of oxidative stress in the testis and epididymis of experimental rats by a herbicide, atrazine. Arch Environ Contam Toxicol.

[5] Kniewald J, Jakominić M, Tomljenović A, Simić B, Romać P, Vranesić D (2000). Disorders of male rats reproductive tract under the influence of atrazine. J Appl Toxicol.

[6] Betancourt M, Reséndiz A, Fierro EC (2006). Effect of two insecticides and two herbicides on porcine sperm motility patterns using computer-assisted semen analyses (CASA) in vitro. Reprod Toxicl.

[7] Stoker TE, Laws SC, Giudici DL, Cooper RL (2000). The effect of atrazine on puberty in male wistar rats: an evaluation in the protocol for the assessment of pubertal development and thyroid function. Toxicol Sci.

[8] Jin Y, Wang L, Fu Z (2013). Oral exposure to atrazine modulates hormone synthesis and the transcription of steroidogenic genes in male peripubertal mice. Gen Comp Endocrinol.

[9] Friedmann AS (2002). Atrazine inhibition of testosterone production in rat males following peripubertal exposure. Reprod Toxicol.

[10] Cooper RL, Stoker TE, Tyrey L, Goldman JM, McElroy WK (2000). Atrazine disrupts the hypothalamic control of pituitary- ovarian function. Toxicol Sci.

[11] Chapin RE, Stevens JT, Hughes CL, Kelce WR, Hess RA, Daston GP (1996). Endocrine modulation of reproduction. Fundam Appl Toxicol.

[12] Sharma PK, Chahuan PK, Fulia A (2011). Atrazine induced morphological alterations in spermatocytes of goat in vitro. J Med Sci.

[13] Feyzi Dehkhargani S, Malekinejad H, Shahrooz R, Sadrkhanloo RA (2011). Detrimental effect of atrazine on testicular tissue and sperm quality: implication for oxidative stress and hormonal alterations. Iranian J Toxicol.

[14] Sahinturk V, Guclu C, Baycu C (2007). Protective effects of vitamin E on ethane dimethane sulfonate-induced testicular toxicity in rats. Asian J Androl.

[15] Momeni HR, Oryan S, Eskandar N (2012). Effect of vitamin E on sperm number and testis histopathology of sodium arsenite- treated rats. Reprod Biol.

[16] Rajabzadeh A, Sagha M, Gholami MR, Hemmati R (2015). Honey and vitamin E restore the plasma level of gonadal hormones and improve the fertilization capacity in noisestressed rats. Crescent J Med Biol Sci.

[17] Khosravanian N, Razi M, Farokhi F, Khosravanian H (2014). Testosterone and vitamin E administration up-regulated varicocele-reduced Hsp70-2 protein expression and ameliorated biochemical alterations. J Assist Reprod Genet.

[18] Willems A, Batlouni SR, Esnal A, Swinnen JV, Saunders PT, Sharpe RM (2010). Selective ablation of the androgen receptor in mouse sertoli cells affects sertoli cell maturation, barrier formation and cytoskeletal development. PLOS One.

[19] O'Donnell L, McLachlan RI, Wreford NG, de Kretser DM, Robertson DM (1996). Testosterone withdrawal promotes stagespecific detachment of round spermatids from the rat seminiferous epithelium. Biol Reprod.

[20] Holdcraft RW, Braun RE (2004). Androgen receptor function is required in sertoli cells for the terminal differentiation of haploid spermatids. Development.

[21] Jaarin K, Gapor MT, Nafeeza MI, Fauzee AM (2002). Effect of various doses of palm vitamin E and tocopherol on aspirin- induced gastric lesions in rats. Int J Exp Pathol.

[22] Minerly AE, Russo SJ, Kemen LM, Nazarian A, Wu HB, Weierstall KM (2008). Testosterone plays a limited role in cocaine-induced conditioned place preference and locomotor activity in male rats. Ethn Dis.

[23] Illingworth PJ, Groome NP, Bryd W, Rainy WE, Mcneilly AS, Mather JP (1996). Inhibin-B: a likely candidate for the physiologically important form of inhibin in man. J Clin Endocrinol Metab.

[24] Benzie IF, Strain JJ (1999). Ferric reducing/antioxidant power assay: direct measure of total antioxidant activity of biological fluids and modified version for simultaneous measurement of total antioxidant power and ascorbic acid concentration. Methods Enzymol.

[25] Hu ML (1994). Measurement of protein thiol groups and glutathione in plasma. Methods Enzymol.

[26] Niehaus WG Jr, Samuelsson B (1968). Formation of malonaldehyde from phospholipid arachidonate during microsomal lipid peroxidation. Eur J Biochem.

[27] Lowry OH, Rosebrough NJ, Farr AL, Randall RJ (1951). Protein measurement with the Folin phenol reagent. J Biol Chem.

[28] Pant N, Srivastava SP (2003). Testicular and spermatotoxic effect of quinaphos in rats. J Appl Toxicol.

[29] Terquem A, Dadoune JP, Andre J (1983). Aniline blue staining of human spermatozoa chromatin: evaluation of nuclear maturation. The sperm cell.

[30] Tejada RI, Mitchell JC, Norman A, Marik JJ, Friedman S (1984). A test for the practical evaluation of male infertility by acridine orange (AO) fluorescence. Fertil Steril.

[31] Kerr JF, Wyllie AH, Currie AR (1972). Apoptosis: a basic biological phenomenon with wide-ranging implications in tissue kinetics. Br J Cancer.

[32] Darzynkiewicz Z (1990). Differential staining of DNA and RNA in intact cells and isolated cell nuclei with acridine orange. Methods Cell Biol.

[33] Skinner MK, Fritz IB (1985). Testicular peritubular cells secrete a protein under androgen control that modulates Sertoli cell functions. Proc Natl Acad Sci USA.

[34] Ashby J, Tinwell H, Steven J, Pastoor T, Breckenridge CB (2002). The effects of atrazine on the sexual maturation of female rats. Regul Toxicol Pharmacol.

[35] Johnson L, Thompson DL Jr, Varner DD (2008). Role of sertoli cells number and function on regulation of spermatogenesis. Anim Reprod Sci.

[36] Russell LD, Peterson RN (1984). Determination of the elongate spermatid-sertoli cell ratio in various mammals. J Reprod Fertil.

[37] Bellvé AR, Zheng W (1989). Growth factors as autocrine and paracrine modulators of male gonadal functions. J Reprod Fertil.

[38] Kniewald J, Peruzović M, Gojmerac T, Milković K, Kniewald Z (1987). Indirect influence of s-triazines on rat gonadotropic mechanism at early postnatal period. J Steroid Biochem.

[39] Agarwal A, Saleh RA (2002). Role of oxidants in male infertility: rationale, significance, and treatment. Urol Clin North Am.

[40] Mishra M, Acharya UR (2004). Protective action of vitamins on the spermatogenesis in lead-treated Swiss mice. J Trace Elem Med Biol.

[41] Oda SS, El-Maddawy ZKh (2012). Protective effect of vitamin E and selenium combination on deltamethrin-induced reproductive toxicity in male rats. Exp Toxicol Pathol.

[42] Kishore RYV, Sreenivasula RP, Shivalingam MR (2010). Protective effects of testosterone on ciplatin induced impairment in spermatogenesis and steroidogenesis in rats. Res J Pharm Technol.

[43] Moss JL, Crosnoe LE, Kim ED (2013). Effect of rejuvenation hormones on spermatogenesis. Fertil Steril.

[44] Kwon YK, Hecht NB (1991). Cytoplasmic protein binding to highly conserved sequences in the 3' untranslated region of mouse protamine 2 mRNA, atranslationally regulated transcript of male germ cells. Proc Natl Acad Sci USA.

